# Squaraine nanoparticles for optoacoustic imaging-guided synergistic cancer phototherapy

**DOI:** 10.1515/nanoph-2023-0358

**Published:** 2023-08-23

**Authors:** Xiao Chen, Xiaopeng Ma, Gui Yang, Guan Huang, Haibing Dai, Nian Liu, Jianbo Yu

**Affiliations:** Longgang Central Hospital of Shenzhen, Shenzhen 518116, China; School of Control Science and Engineering, Shandong University, Jinan 250061, China; PET Center, Department of Nuclear Medicine, The First Affiliated Hospital, Zhejiang University School of Medicine, Hangzhou 310003, China

**Keywords:** squaraine, selenium modulation, nanoparticles, optoacoustic imaging, phototherapy

## Abstract

The unique optical properties of squaraine dyes make them promising for cancer phototheranostics, but the reported squaraines for *in vivo* treatments mainly rely on their photothermal effect, where monotherapy cannot achieve the desired therapeutic effect. Here we generated a type of squaraine capable of killing tumors through both photothermal and photodynamic effects. We optimized squaraine structure with selenium modulation and formulated it into nanoparticles that showed strong absorption of infrared light, negligible fluorescence, good photothermal conversion (66.6 %), and strong photodynamic effects even after several irradiation cycles. In addition, the nanoparticles could be tracked through their strong optoacoustic signal. In mice, the nanoparticles effectively accumulated in tumors and eliminated them upon irradiation, without causing adverse effects. Our work demonstrates the potential of selenium modulation of squaraine for precise cancer diagnosis and treatment through synergistic photothermal and photodynamic effects.

## 1 Introduction

Anti-cancer therapies based on irradiation with near-infrared (NIR) light are attractive because they are non-invasive and can be tightly controlled in space and time, minimizing off-target adverse effects [1–3]. These therapies involve compounds that, after absorption of NIR light, cause local cytotoxic heating (photothermal therapy, PTT) or produce cytotoxic singlet oxygen (photodynamic therapy, PDT) [4–6]. In parallel, these compounds undergo thermoelastic expansion, which can be detected optoacoustically to image the diseased tissues for diagnostics and treatment guidance [7–12]. Among the most promising compounds for cancer phototheranostics are organic chromophores, which have the merits of easy chemical structure tuning, good biocompatibility, and minimal toxicity [13]. For example, the chromophores indocyanine green (ICG) and methylene blue have been licensed for clinical use, but their low photothermal conversion efficiency and poor photostability hampers their theranostic effectiveness.

A more effective chromophore may be squaraine, which features a resonance-stabilized zwitterionic planar structure with an electron-deficient, four-membered ring at its core [14]. Rigidly planar squaraines strongly absorb in the NIR region, leading to high molar absorption coefficients and bright fluorescence useful for biomedical applications [15, 16]. Also, squaraines have been modified in some ways to stabilize them and prevent self-aggregation [13], including chemical modifications of their backbone [17–20] and encapsulation within rotaxanes [21], micelles [22–24], or albumin [25, 26]. These used strategies show their improved photothermal performance, but monotherapy is usually not enough to completely eliminate tumors [2]. Endowing photodynamic property with squraines is a relative easy way to achieve combined therapy. Therefore, structural modification of squaraine backbone is further needed to enhance the synergistic photothermal-photodynamic performance.

**Figure 1:**
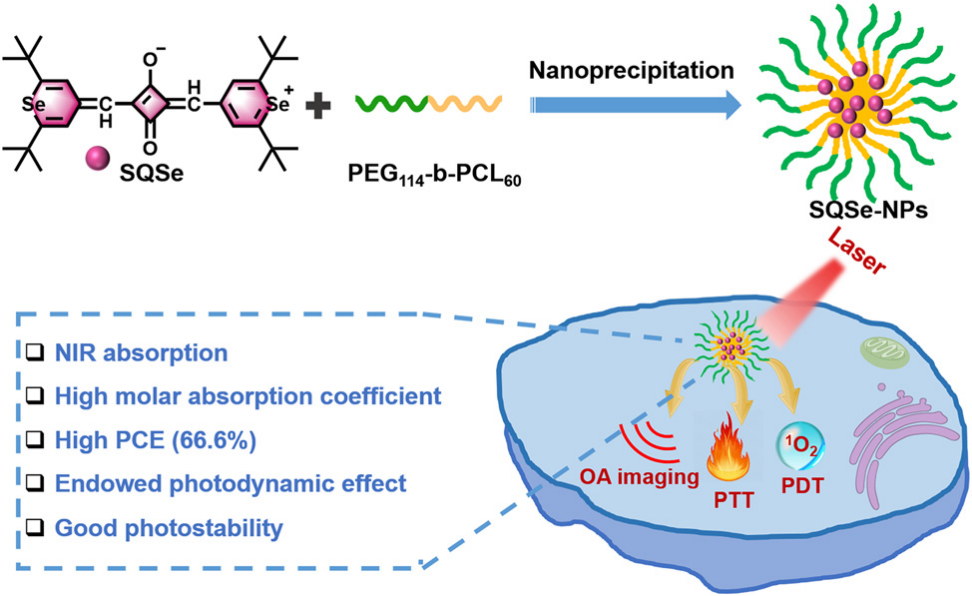
Schematic showing the preparation of SQSe-NPs, and their use as contrast agents for OA imaging and synergistic PTT/PDT.

Heavy atom modulation is used to improve the photodynamic performance of dyes, mainly due to the heavy atom effects causing vibronic spin–orbit coupling of the molecule to increase the probability of intersystem crossing [27, 28]. Herein, we propose to introduce heavy atom (selenium) in the squaraine structure to optimize the optical properties for synergistic photothermal-photodynamic treatments of cancers (Figure 1). We first characterized the physiochemical properties of SQSe and based-nanoparticles (SQSe-NPs) to verify the improved optical and therapeutic performances. Next, we tested the phototoxicity *in vitro*. We further performed the *in vivo* OA imaging to evaluate the optimal time point for treatments. Finally, SQSe-NPs were administrated on tumor-bearing mice to evaluate the therapeutic effect. We expect that SQSe-NPs can be used as a high-efficient photo-theranostic agent for precise cancer treatments.

## 2 Results and discussion

### 2.1 Synthesis and characterization of SQSe

**Figure 2:**
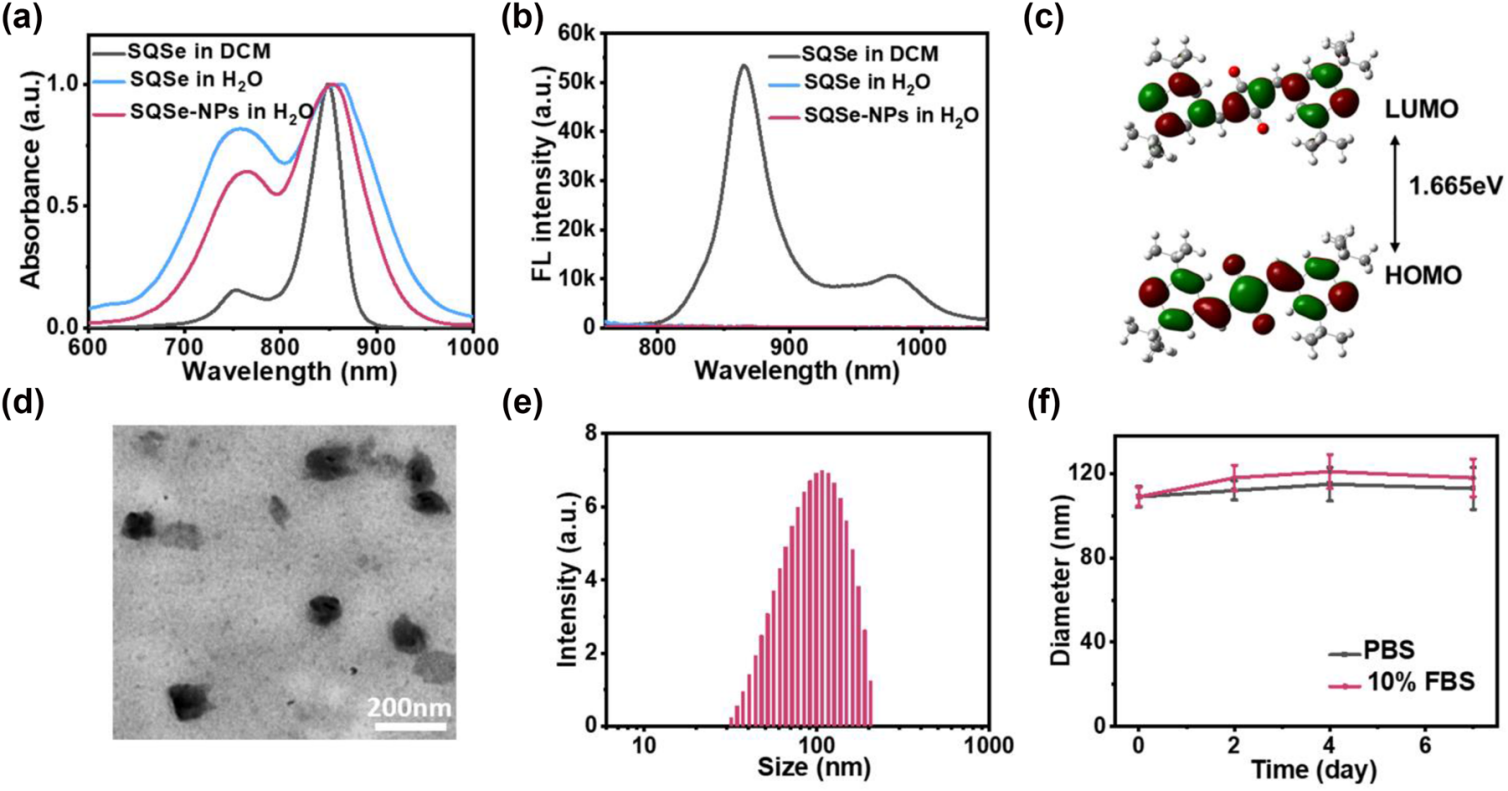
Characterization of SQSe on its own and encapsulated within nanoparticles. (a) Absorption spectra and (b) fluorescence spectra of SQSe in different solvents and SQSe-NPs in water. (c) Distribution of frontier molecular orbitals in SQSe based on density functional theory calculations. The energy gap is shown between the highest occupied molecular orbital (HOMO) and lowest unoccupied molecular orbital (LUMO). (d) TEM image and (e) DLS profile of SQSe-NPs. (f) Estimated diameter of SQSe-NPs during incubation at 4 days in PBS or 10 % FBS.

SQSe was synthesized through condensation of 4-methylchalcogenopyrylium salts with squaric acid (Figure S1), and its chemical structure was verified using MALDI-TOF mass spectrometry, ^1^H NMR, and ^13^C NMR (Figures S2–S4). It showed good solubility in organic solvents. Importantly, SQSe exhibited the enhanced and stable NIR absorption, which is attributed to the acceptor engineering (4-methylchalcogenopyrylium salts) on the electron-deficient squaric acid (Figure 2a). The photophysical properties of SQSe were next characterized. The molar absorption coefficient of SQSe in dichloromethane (DCM) was tested with 2.27 × 10^5^ L mol^−1^ cm^−1^, which is consistent with the reported squaraine dyes [14]. Its quantum yield (Φ = 0.0028) was only 5.6 % that of indocyanine green, which can be attributed to the selenium and which implies that a greater fraction of excitation energy undergoes nonradiative decay and intersystem crossing. To better understand the effect of the SQSe structure on the optical properties, density functional theory (DFT) calculation was performed for SQSe at the B3LYP/Def2SVP level via the Gaussian 16 program package. The highest occupied molecular orbitals (HOMO) are distributed over the conjugated structure, while the lowest unoccupied molecular orbitals (LUMO) localize mainly to acceptor regions. The narrow bandgap between HOMO and LUMO indicated the charge-transfer characteristics of SQSe with longer wavelength absorption (Figure 2c).

### 2.2 Preparation and characterization of SQSe-NPs

To improve the bioavailability of hydrophobic SQSe, we encapsulated it into amphiphilic PEG_114_-b-PCL_60_ to prepare polymeric nanoparticles (SQSe-NPs). We characterized the morphology and size by transmission electron microscopy (TEM) and dynamic light scattering (DLS), which showed around 110 nm of nanosize (Figure 2d and e). The stability of SQSe-NPs was monitored in phosphate-buffered saline (PBS) and 10 % fetal bovine serum (FBS), which showed no significant changes in nanosize (Figure 2f). The optical properties of SQSe-NPs were next determined by absorption spectrum and fluorescence spectrum. Figure 2a showed SQSe-NPs absorbed strongly at 700–900 nm without suffering absorption quenching or blue-shifting. Due to the aggregation-induced quenching effect in nanoformulations, SQSe-NPs have negligible fluorescence (Φ = 0.00004) (Figure 2b).

### 2.3 Photothermal and photodynamic effects of SQSe-NPs *in vitro*

**Figure 3:**
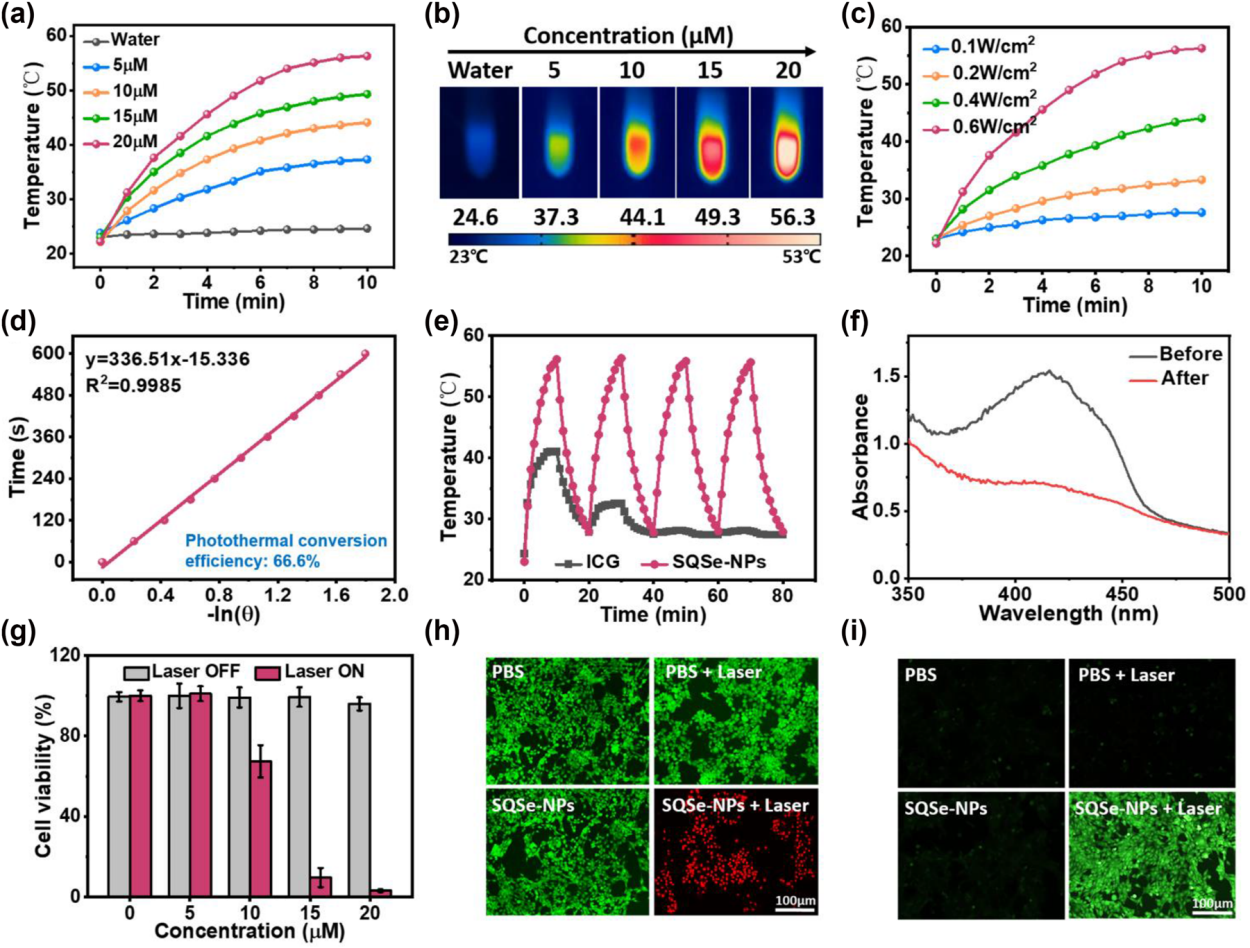
Phototherapeutic effects of SQSe-NPs *in vitro*. (a, b) Photothermal effects and thermal images of SQSe-NPs with various concentrations upon 808 nm laser irradiation (0.6 W/cm^2^). (c) Photothermal effects of SQSe-NPs (20 μM) upon laser irradiation with the indicated power densities. (d) Linear correlation between the cooling time of SQSe-NPs and the negative logarithm of temperature. (e) Photothermal effect of SQSe-NPs during four irradiation cycles. (f) Absorption spectra of DPBF irradiated by 5 min’s 808 nm laser in the presence of SQSe-NPs, the decreased absorption indicated the ROS generation. (g) Relative viability of 4T1 cells exposed to the indicated concentrations of SQSe-NPs. Cultures were exposed or not to 5 min of irradiation with a laser at 0.6 W/cm^2^. (h) Fluorescence images of Calcein-AM (green color, live cells) and propidium iodide (red color, dead cells) co-stained 4T1 cells after various treatments. (i) Fluorescent detection of intracellular ROS after various treatments.

The strong NIR absorption of SQSe-NPs indicated the capacity of NIR light harvesting. To assess the photothermal effect of SQSe-NPs, an 808 nm CW laser was chosen to irradiate various concentrations of SQSe-NPs due to the fact that the absorption of normal tissue and water is lower at this wavelength, which in turn allows the laser to penetrate the tissue to a relatively deeper depth. And the generated heat was monitored by a thermal camera. Figure 3a and b showed the concentration-dependent temperature changes. Under the same laser irradiation, SQSe-NPs (20 μM) can induce huge temperature changes with 34.1 °C while water only has a negligible temperature increase. Such heat generation from SQSe-NPs is sufficient for hyperthermia damage of cancerous cells [3]. Next, we tested the temperature changes of SQSe-NPs with different laser power densities, which showed an obvious laser power intensity dependence (Figure 3c). The photothermal conversion efficiency of SQSe-NPs was calculated as 66.6 %, which was higher than most of the reported squaraines [14, 29, 30] due to favorable electron transfer (Figure 3d). Besides, SQSe-NPs showed a stable temperature increase during 4 cycles of laser irradiation, indicating good photostability (Figure 3e). In addition, the photodynamic effect of SQSe-NPs was also determined by 1,3-Diphenylisobenzofuran (DPBF, a ROS sensor) under the same test tube conditions. The decreased absorption intensity of DPBF means the endowed photodynamic effect of SQSe-NPs, which is attributed to the modulation of selenium (Figure 3f). The singlet oxygen quantum yield of SQSe-NPs was determined to be ∼0.03 using ICG as a reference [31].

We next evaluated the *in vitro* phototherapeutic effect on 4T1 breast cancer cells. We used the Cell Counting Kit-8 (CCK8) assay to evaluate the photo-cytotoxicity of SQSe-NPs on these cells after 8 h’s incubation. SQSe-NPs showed negligible cytotoxicity even at a concentration of up to 20 μM (Figure 3g). However, upon laser radiation, the cell viability decreased dependent on SQSe-NPs concentration and was nearly 0 % when treated with 20 μM of SQSe-NPs. Next, the pure PDT or PTT effects of SQSe-NPs was investigated by ice water bath to obstruct heat generation or adding glutathione (GSH) to eliminate ROS production. Figure S5 showed that monotherapy had partial inhabitation of cancerous cells growth while synergistic phototherapy had the improved celling killing. To directly visualize the phototherapeutic effect of SQSe-NPs on 4T1 cells, calcein-acetoxymethyl (calcein-AM) and propidium iodide were co-stained with the treated cells to distinguish between live and dead cells. Figure 3i showed the strong photo-cytotoxicity on SQSe-NPs + Laser treated cells while minimizing damage in the control groups. To further explore the photodynamic effect of cancerous cells, 2′,7′-dichlorodihydrofluorescein diacetate (DCFH-DA) was co-incubated with the treated cells to visualize the ROS generation upon laser irradiation. Figure 3h displayed strong green fluorescence from SQSe-NPs + Laser treated cells but few weak signals from the other 3 control groups.

### 2.4 OA imaging of SQSe-NPs in phantoms and *in vivo*

**Figure 4:**
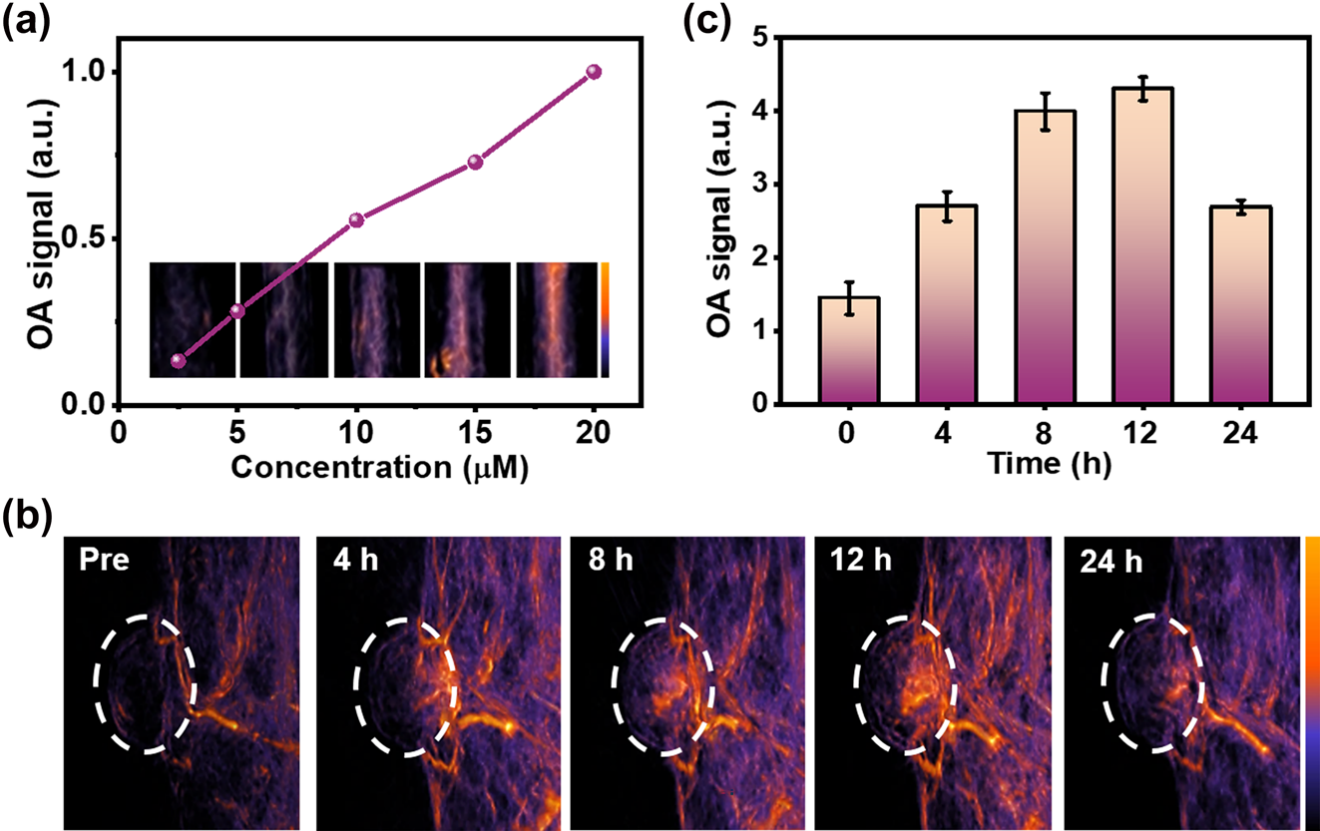
OA imaging of SQSe-NPs. (a) OA signals of the indicated concentrations of SQSe-NPs in water in a thin tubing. (b) Representative OA images of 4T1 tumor-bearing mice at different time points after injection of SQSe-NPs, the white circle indicated the tumor area. (c) Quantitation of OA signals in tumors from the experiment in panel (b).

To examine the *in vitro* OA performance of SQSe-NPs, we first tested them with various concentration gradients, which is a good linear fit between the OA signals and concentrations (Figure 4a). Given the promising optoacoustic (OA) properties of SQSe-NPs, 4T1 tumor mouse models were established to explore the possibility of using SQSe-NPs for tumor detection *in vivo*. Figure 4b and c showed representative 3D OA images of 4T1 tumor-bearing mice at various time points postinjection of SQSe-NPs. It is obvious to see that SQSe-NPs were progressively accumulated in the tumor region due to the enhanced permeability and retention effect. The OA intensity of the tumor reached its maximal accumulation between 8 and 12 h of administration, which can be considered the optimal time point for cancer phototherapy. Subsequently, the OA signals of tumor areas were decreased due to systemic clearance from the tumor vasculature. After 24 h of intravenously injection, the major organs and tumor were isolated and done the OA measurements. Except tumor retention, SQSe-NPs showed higher accumulation in liver and kidney (Figure S6).

### 2.5 Phototherapeutic effect of SQSe-NPs *in vivo*

**Figure 5:**
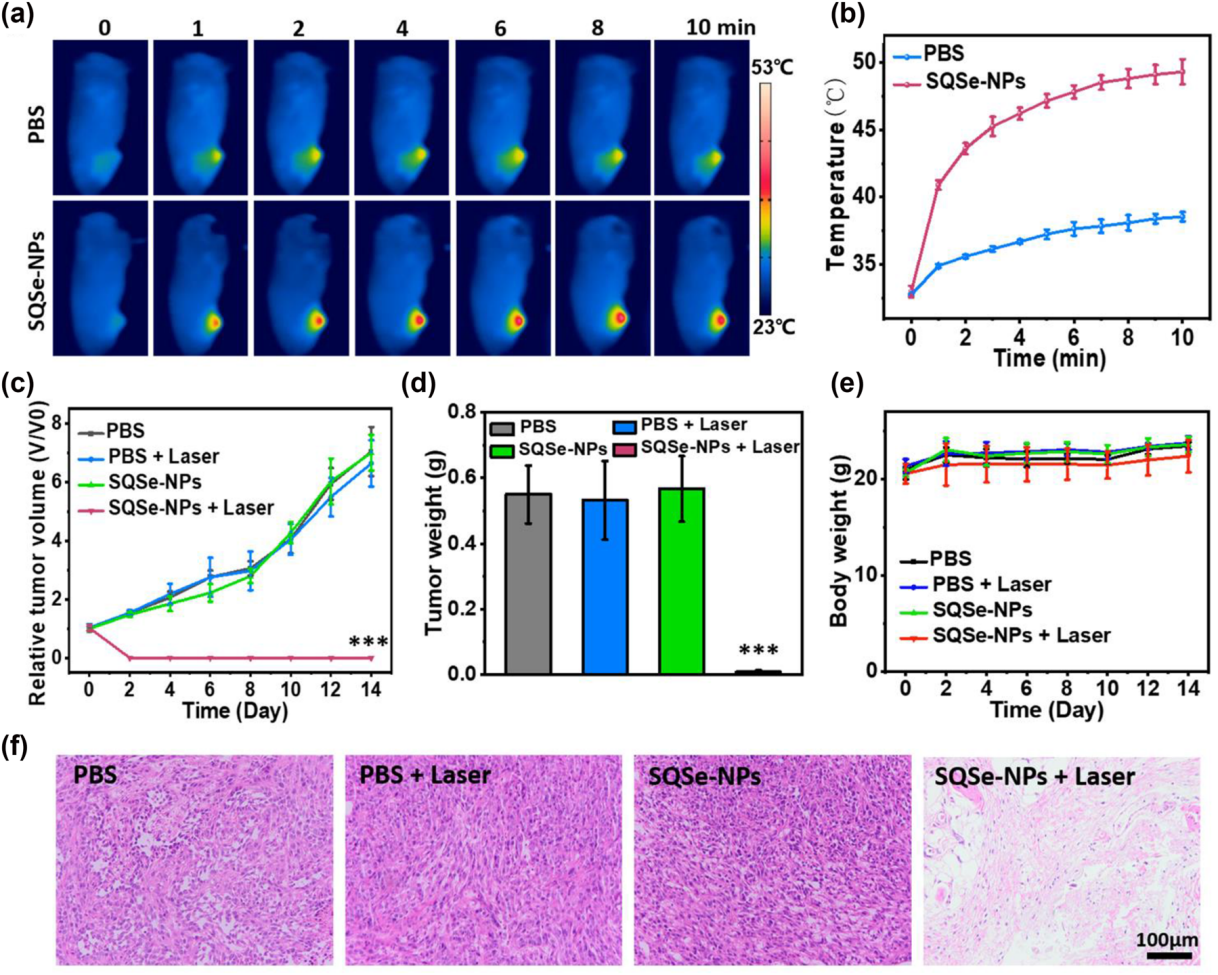
Phototherapeutic effects of SQSe-NPs *in vivo*. Mice bearing 4T1 breast cancer xenograft tumors were treated with PBS or SQSe-NPs, then irradiated or not with an 808 nm CW laser. (a) Thermal images of laser-irradiated 4T1 tumor-bearing mice with PBS or SQSe-NPs administrations (*n* = 5). (b) Temperature changes during the time course shown in panel (a). (c–e) Tumor growth, tumor weight, and body weight of the treated mice during 14 days’ observations. (f) H&E stained images of tumor slices from the different groups.

Considering the promising photothermal and photodynamic effects of SQSe-NPs on cell killing, we performed to test of the phototherapy on mice bearing 4T1 tumor xenograft. With regard to this, 4T1 tumor-bearing mice were randomly divided into 4 groups (*n* = 5 each) with the following treatments respectively: PBS, PBS + laser, SQSe-NPs, SQSe-NPs + laser. The irradiation time was operated at 8 h post-injection based on the *in vivo* OA imaging results. The temperature changes in tumor areas were monitored by a thermal imaging camera for 10 min. Figure 5a and b shows that the tumor temperature from SQSe-NPs treated mice increased to 49.3 °C, while the control group only have 38.5 °C. Subsequently, H2DCFDA was intratumorally injected into the 4 groups to validate the synergistic phototherapy effect. The *ex vivo* tumors from the treatment of SQSe-NPs + laser showed the excited fluorescence, indicating the *in vivo* synergistic photodynamic effect (Figure S7). The animals’ tumor volumes and body weights were measured every 2 days until 14 days. The mice with the treatment of SQSe-NPs + laser showed complete tumor eradication, while the control groups displayed rapid tumor growth with necrosis, suggesting efficient cancer treatment using SQSe-NPs (Figure 5c and d, S8). Besides, hematoxylin and eosin (H&E) staining was further used to confirm the therapeutic effect on tumors. The tumor slices from treated mice showed cell shrinkage and nucleus fragmentation with severe necrosis due to the phototherapeutic effect, while the other control groups displayed a high number of abnormal cells with typical cancerous features (Figure 5f). In addition, the biosafety of SQSe-NPs was evaluated by body weight monitoring and H&E staining of major organs. The body weight of all the animals showed a normal variation during the observation period, suggesting the absence of any serious side-effects (Figure 5e). H&E staining did not find major signs of toxicity, confirming the promising biocompatibility of SQSe-NPs (Figure S9). Overall, these results indicate that SQSe-NPs have excellent phototherapeutic performance on tumors with good biosafety.

## 3 Conclusions

Here we employed the strategy of selenium modulation to optimize squaraine structure, producing a molecule that strongly absorbs near-infrared wavelengths and efficiently converts excitation energy into local heating and production of reactive oxygen species. A formulation of SQSe into hydrophilic nanoparticles led to efficient tumor killing in a mouse model of cancer, and the nanoparticles could be imaged non-invasively by optoacoustics. Our results establish a new reagent with potential for cancer phototheranostics, and more generally they demonstrate the potential of doping organic dyes with heavy metals for creating strong photothermal and photodynamic effects.

## 4 Methods

### 4.1 Materials

All chemical agents were purchased from Aladdin Scientific Ltd. (Beijing, China). PEG_114_-b-PCL_60_ was ordered from Tanshui Technology Co., Ltd (Guangzhou, China). The Cell Counting Kit-8 (CCK8), 1,3-Diphenylisobenzofuran (DPBF), 2′,7′-dichlorodihydrofluorescein diacetate (H2DCFDA), calcein acetoxymethyl ester (Calcein-AM), and propidium iodide were bought from Beyotime Biotechnology Ltd. (Shanghai, China).

### 4.2 Synthesis of SQSe

SQSe was synthesized by a condensation reaction [32] of squaric acid and 2,6-Di-tert-butyl-4-methylseleninium hexafluorophosphate. Briefly, squaric acid (14 mg, 0.12 mmol, 1 eq) was dissolved with 2,6-di-tert-butyl-4-methylseleninium hexafluorophosphate (100 mg, 0.24 mmol, 2 eq) in methanol (2 mL) under argon, then pyridine (20 mg, 0.24 mmol, 2 eq) was added dropwise. The mixture was incubated at 100 °C for 3 h, then allowed to cool to room temperature. The crude product was concentrated under reduced pressure, then purified by chromatographed (silica gel, DCM/MeOH = 50:1) to yield SQSe (10 mg, 13 %). MALDI-TOF MS calculated for C_32_H_42_O_2_Se_2_ *m*/*z* 618.15, found 615.967. ^1^H NMR (500 MHz, CDCl_3_) *δ* = 9.20 (s, 2H), 6.97 (s, 2H), 6.23 (s, 2H), 1.46 (s, 18H), 1.34 (s, 18H). ^13^C NMR (126 MHz, CDCl3) *δ* = 184.27, 177.01, 166.25, 166.09, 148.93, 125.99, 125.71, 115.40, 40.86, 39.86, 31.23, 31.10.

### 4.3 Synthesis of SQSe-NPs

SQSe (1 mg) and PEG_114_-b-PCL_60_ (4 mg) were dissolved in DMSO (1 mL), then added dropwise to deionized water (15 mL), which was left stirring for 1 h. The suspension was concentrated on a microcentrifuge filter with a molecular weight cut-off of 100 kDa to obtain the purified SQSe-NPs were obtained for further use.

### 4.4 Characterization

^1^H and ^13^C NMR spectra were recorded on Bruker 500 Fourier transform spectrometer. Mass spectrometry was determined using a MALDI UltrafleXtreme (Bruker) with a matrix of dihydroxybenzoic acid. The molar absorption coefficient and relative quantum yield of dyes were calculated as previously described, where ICG in ethanol is as the reference solution (0.05) [33]. Nanoparticles were characterized using a transmission electron microscopy (FEI Tecnai F20) and a dynamic light scattering instrument (Malvern Zetasizer). Absorption spectra were recorded on a SHIMADZU UV-3600 Plus UV-Vis-NIR spectrometer. Fluorescent spectra were recorded in a steady state and time-resolved modes using photoluminescence spectrometer (Edinburg FLS1000).

### 4.5 Analysis of photothermal and photodynamic effects of SQSe-NPs

SQSe-NPs in phantoms were irradiated with an 808 nm continuous-wave laser (Changchun New Industries Optoelectronics Technology Co., Ltd., 0.6 W/cm^2^), and the temperature was measured using an infrared thermal camera. The photothermal conversion efficiency (PCE) of SQSe-NPs was calculated as described [34] using the equation *η* = [(hS(T − Tsurr) − QDis]/I(1−10^−A808^), where A808 refers to the absorbance of SQSe-NPs at 808 nm (0.58) and I refer to the laser power (0.6 W/cm^2^). The photostability of SQSe-NPs was assessed by several cycles of irradiation to check if the temperature changes can remain the same. The same excitation was performed in the presence of DPBF, a sensor of reactive oxygen species, in order to assess photodynamic performance and determine ^1^O_2_ quantum yield [31]. In this assay, generation of singlet oxygen leads to a decrease in absorption.

### 4.6 Photocytotoxicity of SQSe-NPs *in vitro*

4T1 cells (1 × 10^4^ per well) were subcultured in a 96-well plate overnight and treated with various concentrations of SQSe-NPs for 8 h. Then cultures were irradiated or not for 5 min with an 808 nm CW laser (0.6 W/cm^2^) and incubated overnight. Cultures were assessed for viability using the CCK8 assay. The live/dead cells assays were used to visualize the phototherapeutic effect at the cellular level, where the treated cells were co-stained with Calcein-AM and propidium iodide for 30 min and then imaged using Olympus BX53 Upright Fluorescence Microscope. The *in vitro* photodynamic effect SQSe-NPs was identified by H2DCFDA. Briefly, the cells were incubated with 8 h’s SQSe-NPs and another 1 h’s H2DCFDA, and then the cells were replaced with fresh medium and done with 5 min’s laser irradiation. The photodynamic effect of SQSe-NPs at the cellular level was visualized by Olympus BX53 Upright Fluorescence Microscope.

### 4.7 Xenograft tumor models

All animal protocols were approved by the Animal Care and Use Committee of the First Affiliated Hospital of Zhejiang University School of Medicine. 4T1 breast cancer cells were subcutaneously injected into the right legs of female BALB/c mice (6–8 weeks). Approximately 1 × 10^6^ cells were injected into each animal. When tumors had grown to a volume of 100 mm^3^, animals were used for further experiments (see below).

### 4.8 OA imaging of SQSe-NPs *in vitro* and *in vivo*

The OA imaging studies were operated on a LOIS-3D Pre-Clinical Mouse Imaging System (TomoWave, USA) with 808 nm-fixed pulsed laser and ∼150 μm of spatial resolution. Mice were intravenously injected with SQSe-NPs (0.3 mM, 150 μL), then imaged at various time points (4 h, 8 h, 12 h, 24 h). Animals were also imaged immediately before injection of SQSe-NPs. OA signals were quantitated using 3D Slicer software.

### 4.9 Phototherapy with SQSe-NPs in 4T1 tumor-bearing mice

At 8 h after injection of SQSe-NPs (0.3 mM, 150 μL) or phosphate-buffered saline (PBS) as a negative control, animals were irradiated or not for 10 min at 808 nm (0.6 W/cm^2^). Five mice were subjected to each treatment. Tumor temperature was recorded using a thermal camera. Tumor volume and body weight of mice were recorded every two days. After 14 days of observation, all mice were sacrificed and the vital organs and tumors were isolated for histology based on staining with hematoxylin and eosin (H&E).

### 4.10 Statistical analysis

Statistical analysis was performed using Origin 2021. Inter-group differences were assessed for significance using One-Way/Two-Way ANOVA with Tukey’s HSD test. Results were expressed as mean ±SD, and differences were considered significant if *P* < 0.05.

## Supplementary Material

Supplementary Material Details
