# Supplementary material for: Squaraine nanoparticles for optoacoustic imaging-guided synergistic cancer phototherapy
Source: Nanophotonics. 2023 Aug 23;12(18):3645–52. doi: 10.1515/nanoph-2023-0358 (PMC11501745; doi:10.1515/nanoph-2023-0358)
Supplement: Supplementary file 1 — Supplementary Material Details [file j_nanoph-2023-0358_suppl_001.docx]

Supporting Information

**Squaraine nanoparticles for optoacoustic imaging-guided synergistic cancer phototherapy**

Xiao Chen, ^†^ Xiaopeng Ma,^‡^ Gui Yang,^†^ Guan Huang,^†^ Haibing Dai,^†^ Nian Liu,^§,^* Jianbo Yu^†,^*

^†^ Longgang Central Hospital of Shenzhen, Shenzhen 518116, China

^‡^ School of Control Science and Engineering, Shandong University, Jinan 250061, China.

^§^ PET Center, Department of Nuclear Medicine, The First Affiliated Hospital,
Zhejiang University School of Medicine, Hangzhou 310003, China.

^*^Correspondence: nian.liu@zju.edu.cn, jianboyu2001@sohu.com

^
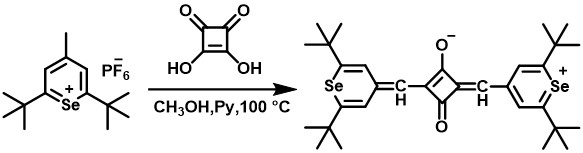
^

**Figure S1.** Synthesis procedures of SQSe.


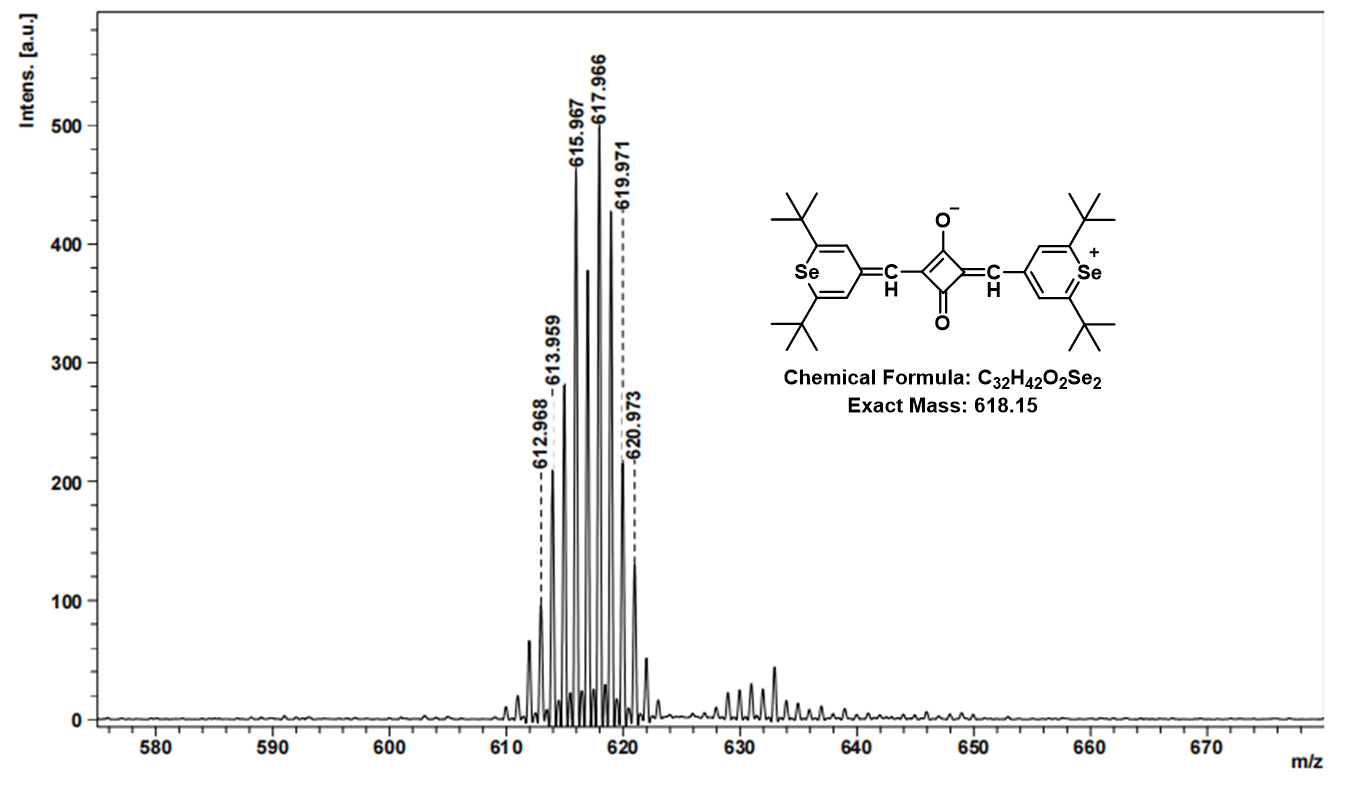


**Figure S2.** MALDI-TOF spectrum of SQSe.


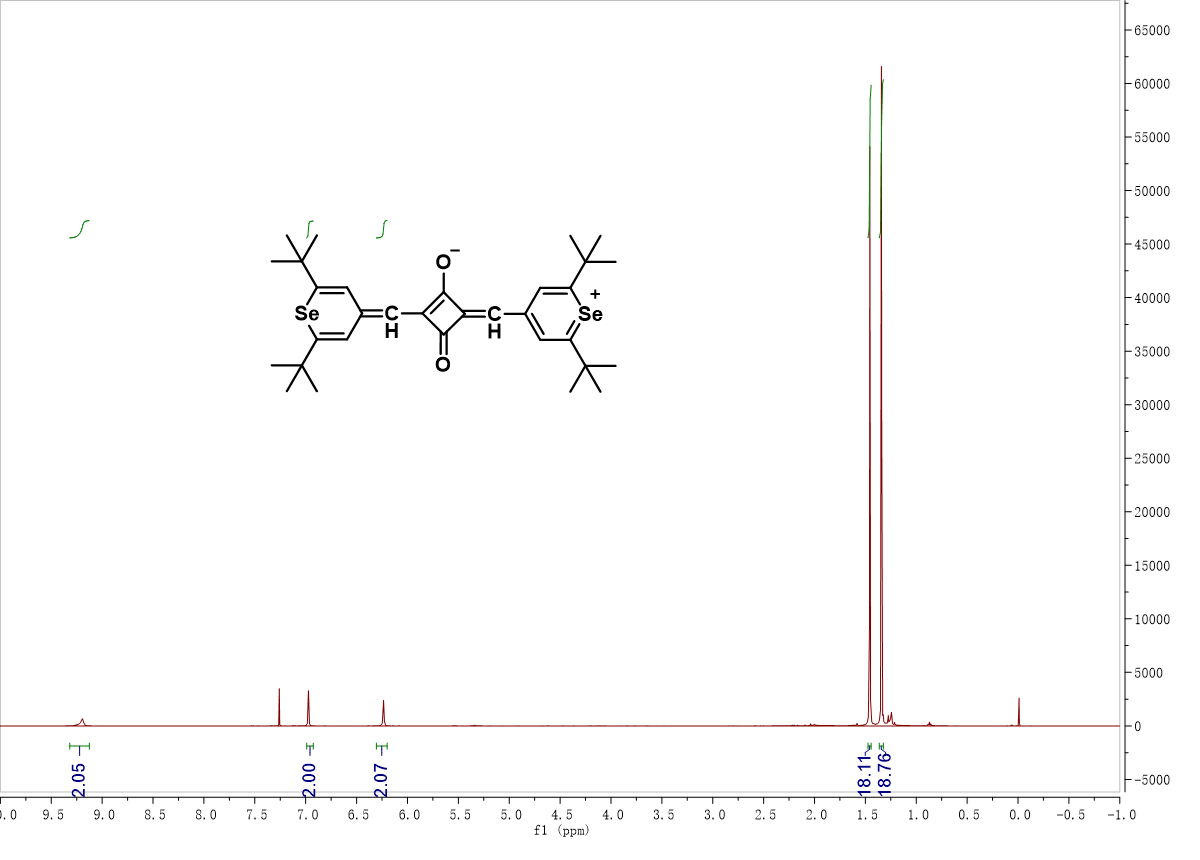


**Figure S3**. ^1^H NMR spectrum of SQSe.


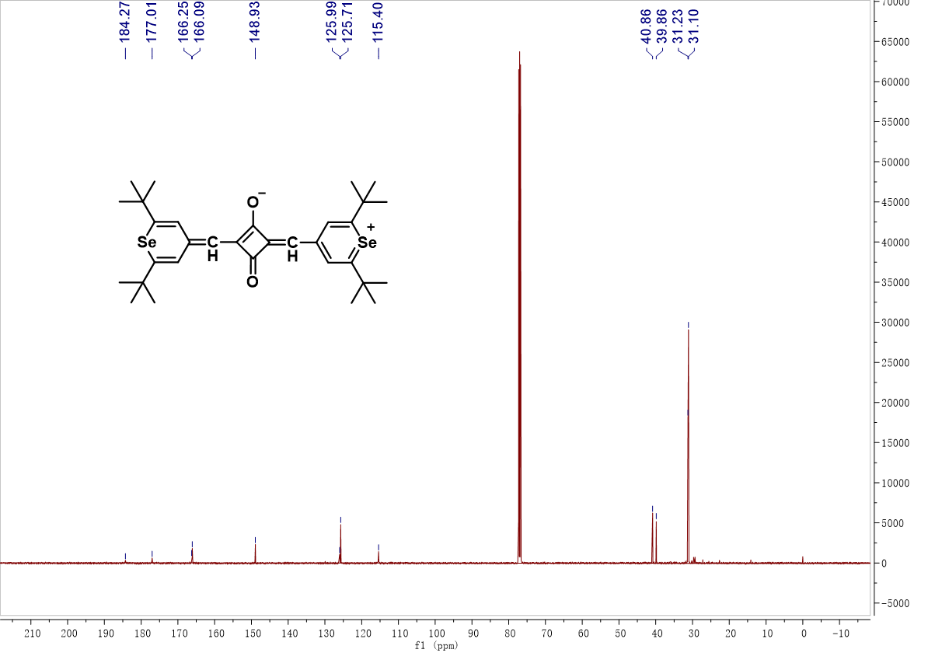


**Figure S4**. ^13^C NMR spectrum of SQSe.

**Figure S5**. Relative viability of 4T1 cells with various treatments. Control: cells without any treatment; PTT: incubate with 15 μM SQSe-NPs and GSH (100 μM antioxidant), following by irradiation of an 808 nm CW laser (0.6 W/cm^2^); PTT: incubate with 15 μM SQSe-NPs, following by irradiation of an 808 nm CW laser (0.6 W/cm^2^) with ice water bath; PTT&PDT: incubate with 15 μM SQSe-NPs, following by irradiation of an 808 nm CW laser (0.6 W/cm^2^).


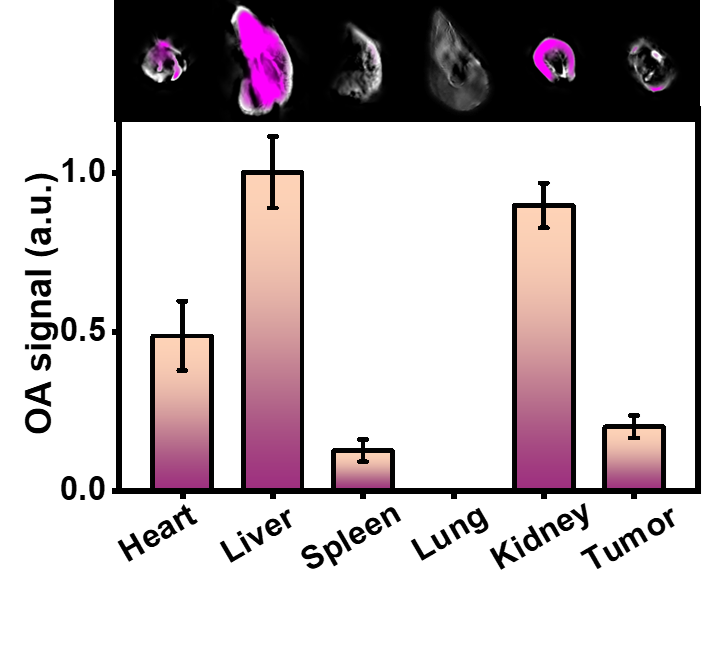


**Figure S6**. OA coronal plane images of major organs and tumor at 24 h post-injection and corresponding OA signal intensities.


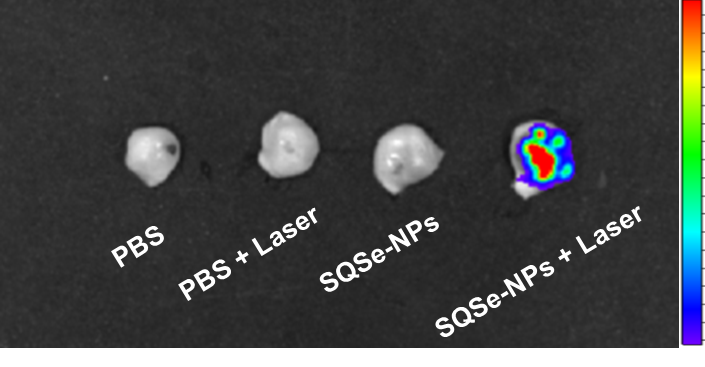


**Figure S7**. The representative IVIS fluorescence images of *ex vivo* tumors from different group. The mice were administrated with various treatments and then intratumorally injected of H2DCFDA, respectively. The excited fluorescence means the ROS generation *in vivo*.


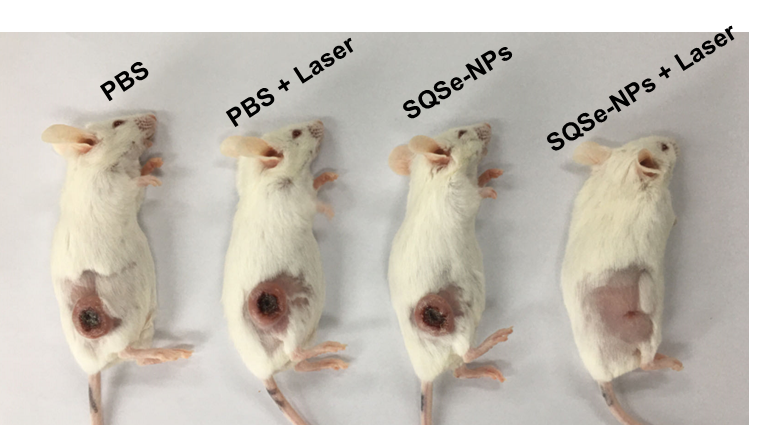


**Figure S8**. Photograph of tumor-bearing mice from each group at day 14 post-treatment.


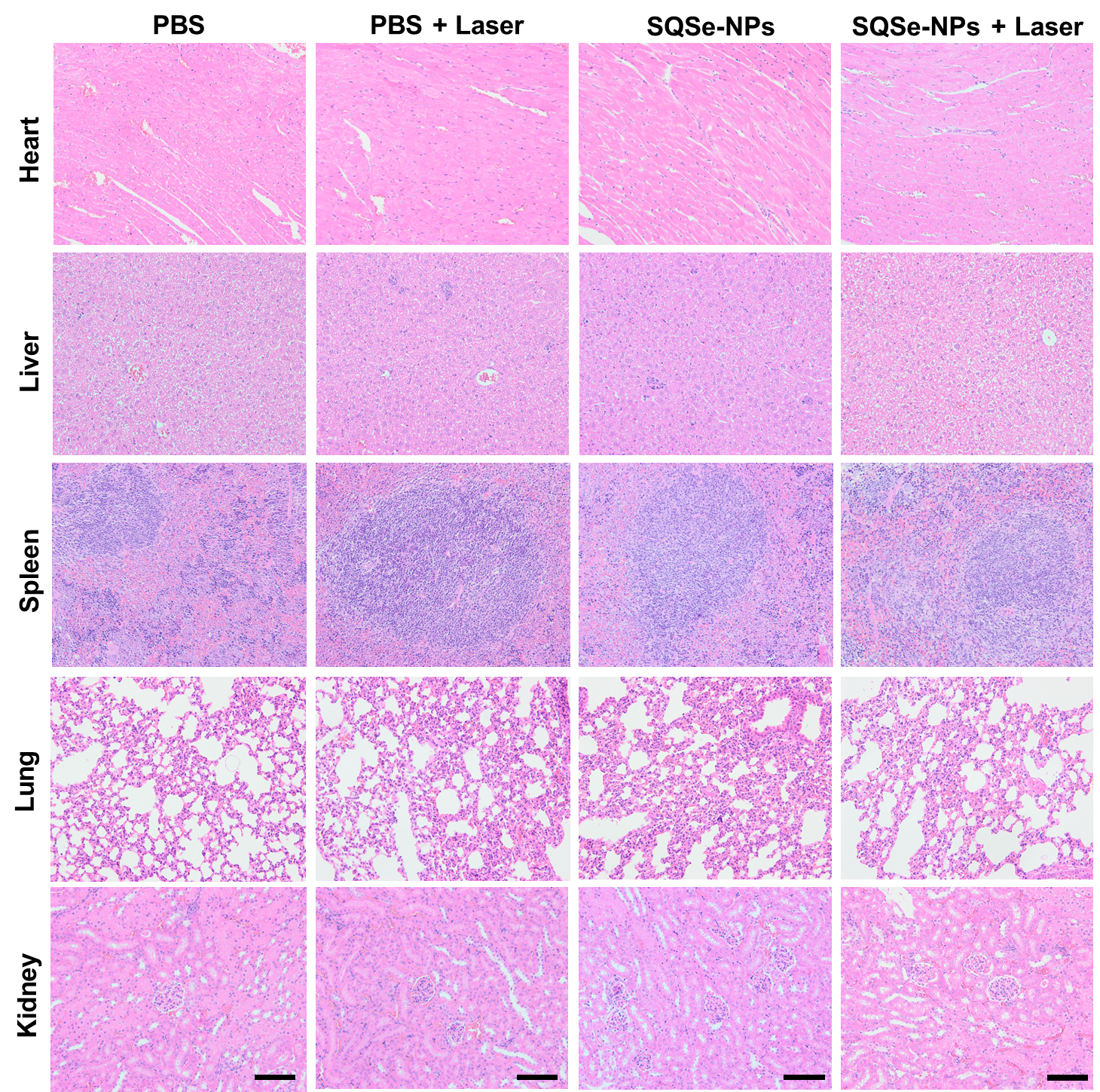


**Figure S9.** H&E stained images of vital organs with various treatments. Scale bar = 100 μm.
